# Deep calibration of financial models: turning theory into practice

**DOI:** 10.1007/s11147-021-09183-7

**Published:** 2021-08-17

**Authors:** Patrick Büchel, Michael Kratochwil, Maximilian Nagl, Daniel Rösch

**Affiliations:** 1 Commerzbank AG, Mainzer Landstraße 157, 60327 Frankfurt am Main, Germany; 2 Dr. Nagler & Company GmbH, Maximilianstraße 47, 80538 Munich, Germany; 3 Universtät Regensburg, Chair of Statistics and Risk Management, Universitätsstraße 31, 93040 Regensburg, Germany

**Keywords:** Deep learning, Derivatives, Model calibration, Interest rate term structure, Global optimizer

## Abstract

The calibration of financial models is laborious, time-consuming and expensive, and needs to be performed frequently by financial institutions. Recently, the application of artificial neural networks (ANNs) for model calibration has gained interest. This paper provides the first comprehensive empirical study on the application of ANNs for calibration based on observed market data. We benchmark the performance of the ANN approach against a real-life calibration framework that is in action at a large financial institution. The ANN based calibration framework shows competitive calibration results, roughly four times faster with less computational efforts. Besides speed and efficiency, the resulting model parameters are found to be more stable over time, enabling more reliable risk reports and business decisions. Furthermore, the calibration framework involves multiple validation steps to counteract regulatory concerns regarding its practical application.

## Introduction

The calibration of financial models is a laborious, time-consuming and expensive task performed by financial institutions on a regular basis (e.g., daily). Asset pricing models are used to determine the value of derivatives or to generate scenarios for Monte Carlo calculations in risk management. Hence, the outcomes of these models are crucial information required for investment and business decisions. The calibration of these models needs to be performed frequently to ensure the validity of their outcomes. In particular, the calibration of complex and multi-dimensional models is burdensome and requires significant computational efforts and time. The choice of an asset pricing model for a specific product involves balancing the accuracy of the model and the time required for its calibration.

Calibration of a financial model can be described as a reverse optimization task, where the inputs of a pricing function (model parameters) are determined to fit observable outputs (e.g., market prices). The solution of this problem usually requires calling a specific pricing function a large number of times with different parameter settings. Hence, the required time and computational resources have always been limiting factors when choosing a pricing model and models with fast (semi-)analytical solutions are generally preferred. Furthermore, these limitations have led to the broad application of local optimization algorithms for calibration, see Liu et al. (2019). The application of more advanced optimization algorithms is rarely considered. Particularly models with multiple parameters give rise to multiple minima for calibration. Hence, local optimization algorithms tend to struggle finding a robust solution.

Given the aforementioned issues and limitations, the application of machine learning for the calibration of asset pricing models has recently gained interest. In particular, the application of artificial neural networks (ANNs) for accelerating the pricing of derivatives is a topic of interest. As one of the first, Hutchinson et al. (1994) analyzed the applications of ANNs to estimate the pricing function for derivatives in a non-parametric, model-free way. This idea was resumed amongst others by Quek et al. (2008) and Culkin and Das (2017).^1^ Recently various papers emerged dealing with a model-based approximation of derivative pricing functions under advanced asset pricing models. For example, Ferguson and Green (2018) apply a forward feed network to estimate the valuation function for equity basket options. Hirsa et al. (2019) analyse the performance of ANN pricing methods for European, Barrier and American options under different mathematical regimes. Liu et al. (2019) use ANNs for the approximation of option values under the Black & Scholes and Heston model. With respect to interest rate models, Kienitz et al. (2020) analyze the application of ANNs for the approximation of swaption prices under the Hull-White and Trolle–Schwartz model.

Based on the application of ANNs for the pricing of derivatives, there are several papers on utilizing these trained ANNs for calibration. Hernandez (2017) firstly presented this idea by applying a feed forward ANN for the calibration of a single-factor Hull-White model based on real market data (Sterling ATM swaptions). Dimitroff et al. (2018) use convolutional neural networks for the calibration of stochastic volatility models. As the application of ANNs is expected to accelerate the pricing process, the application of more complex models is an intensively discussed issue. In particular, the calibration of rough volatility models is extensively analyzed by Bayer and Stemper (2018), Bayer et al. (2019), Horvath et al. (2021) and Stone (2020) . The general idea is the acceleration of the instrument valuation via the application of a neural network. The optimization itself is in most cases still based on a local optimization algorithm. Furthermore, most of the existing papers do not use real market data to assess the performance of the ANN, but use only simulated data. Correspondingly, there is no study which compares the ANN results to a real-life implementation at a financial institution to shed light on practical benefits.

We employ the calibration framework proposed by Liu et al. (2019). It involves a two-step procedure for the calibration of financial models. First, a feed forward ANN is trained based on simulated training data to approximate the valuation function under a given asset pricing model.^2^ Second, the trained ANN is utilized in a backward manner for the calibration of model parameters. We apply the calibration framework to an interest rate (IR) term structure model based on Trolle and Schwartz (2009), as this setup is applied in the benchmark implementation.

While Liu et al. (2019) show the effectiveness of their approach on simulated data for the training of the ANN as well as the calibration of the model parameters, we empirically analyze the performance of this framework based on a comprehensive set of historic market data for a consecutive series of trading days (21 months). Hernandez (2017) uses historic market data for the calibration of the Hull-White model, but the data is limited to ATM swaptions. Furthermore, the adjustments to the Hull-White model, such as keeping the parameters constant across swaption maturities are considered as being too simplistic for practical application (Kienitz et al. (2020)). Hence, we consider our study as the first comprehensive empirical assessment that deeply examines the application of ANNs for calibration of financial models based on real market data. The purpose of the paper is to answer the question if current calibration frameworks of financial institutions can be accelerated, maintaining similar calibration accuracy. This would make it possible to use more advanced financial models or/and optimizers for the calibration tasks frequently performed by risk managers.

We extend the literature regarding the calibration of IR term structure models in three important ways. We are the first to establish an ANN for the valuation of swaptions under the Trolle–Schwartz (TS) model and validate the results based on historical market data, evaluating their performance in real-life situations. Second, we calibrate the Trolle–Schwartz model parameters for a consecutive series of trading days based on historic market data for EUR swaptions using a global optimization algorithm. We find that the resulting model parameters using a global optimizer are more stable compared to the benchmark implementation which is in action at a large financial institution. This has important managerial implications as more stable parameters might contribute to less volatile P&L figures over time, which is a desirable outcome for financial institutions. Furthermore, several more simplistic but widely used IR term structure models can be recovered from the Trolle–Schwartz model by using assumptions for certain parameters (Trolle and Schwartz 2009). Therefore, we consider our results interesting not only for institutions using the TS model, but for a wide range of market participants applying less complex IR term structure models. Third, we outline lessons learned for the practical application of ANNs for financial model calibration and decision making in risk management.

The rest of the paper is structured as follows. In Sect. 2, we briefly introduce the Trolle–Schwartz model and show the procedure for calibrating the model. Section 3 provides a detailed explanation of the ANN calibration approach and its subsequent components. The data, methodology and results of our comprehensive empirical study are presented in Sect. 4. This includes the validation and benchmarking of our results. Section 5 concludes this paper.

## Calibration of interest rate term structure models

### The benchmark implementation

The calibration of interest rate term structure models is a widely faced task in the financial industry. In general, more complex models are accompanied by higher computational burden and an increase of time required for calibration. Therefore, financial institutions usually set up a costly infrastructure for the calibration of these financial models. However, they have to find a trade-off between the complexity of a financial model, the optimization algorithms and the available time in their daily calibration task. Hence, the computational resources are a limiting factor, when choosing pricing models and optimization algorithms. We set out to validate the ANN approach on empirical data and benchmark against a traditional calibration framework which is in action at a large financial institution. The traditional framework uses a semi-analytical solution of the Trolle–Schwartz model for the pricing of European swaptions, when performing the calibration task. The daily calibration at the financial institution is processed on a large computing cluster utilizing 72 CPU cores simultaneously. Due to time constraints in the productive setting, a local optimizer is used. This is called the “benchmark implementation” henceforth. To make a fair comparison, we use the exact same set of instruments and the same calibration loss function. The aim of the following sections is to show if an ANN can accelerate and increase the robustness of calibration frameworks at financial institutions, while maintaining similar calibration results.

### Model calibration

The calibration of financial models is a reverse optimization problem. We assume that we can use a given model to calculate prices of certain financial instruments. The calculation of the price estimate ($\hat{p}_j^{(model)}$) under a specific model for a given instrument (*j*) requires a series of inputs. This includes the properties of the instrument ($\tau _j$), the parameters of the model ($\Omega _t =(\omega _{t1},\ldots ,\omega _{tp})$), where *p* is the number of parameters to calibrate, and a set of market data ($\Lambda _t$) at a specific point in time (*t*). By applying a calibration procedure, the model parameters are set such that the difference between the resulting model prices and the observable market prices is minimized given a specific loss function (L):

1 $$\begin{aligned} {{\,\mathrm{arg\,min}\,}}_{\Omega _t} \sum _{j\in \mathscr {F}_t} L\left( p_j^{(market)},\hat{p}_j^{(model)}(\Omega _t \ |\ \tau _j,\Lambda _t)\right) , \end{aligned}$$

where $\mathscr {F}_t$ represents a set of financial instruments, which have observable market prices ($p_j^{(market)}$). The calibration requires a reasonable and thoughtful choice of calibration instruments. Instruments used for calibration should be liquid, frequently traded and inherit all relevant risk drivers of the instruments it will be applied to. Furthermore, the quality of the calibration is limited by the ability of the model to capture all relevant risk drivers and dependencies of the observable market prices. Nevertheless, the calibration of a complex and high-dimensional model might be quite burdensome from a methodological and computational point of view. Hence, the choice of an appropriate model requires balancing accuracy and computational performance. Especially, if these models are used for pricing financial instruments the ability to perform the calibration in a reasonable amount of time is a crucial prerequisite for their practical application, e.g., for investment or hedging decisions. In addition, the traceability and interpretability of the model is an important feature and considered a key aspect in supervisory oversight and validation.

### The Trolle–Schwartz model

In this paper, we perform an empirical study for the application of an ANN based framework to calibrate an interest rate term structure model. We use a term structure model based on Trolle and Schwartz (2009), the so called Trolle–Schwartz model (TS henceforth), used by the real-life benchmark implementation. The TS model is an advanced stochastic volatility model based on the Heath-Jarrow-Morton framework (Heath et al. 1992). We use the TS model in its risk-neutral setting. The TS model consists of two stochastic processes for the instantaneous forward rate and the variance of the rate process. The dynamics of the forward rate are modelled as follows (see Trolle and Schwartz 2009):^3^

2 $$\begin{aligned} df(t,T)= & {} \mu _f(t,T)dt + \sum _{i=1}^{N}\sigma _{f,i}(t,T)\sqrt{v_i(t)}dW_i^{\mathbb {Q}}(t) \end{aligned}$$

3 $$\begin{aligned} dv_i(t)= & {} \kappa _i\left( \theta _i-v_i(t)\right) dt+\sigma _i\sqrt{v_i(t)}\left( \rho _idW_i^{\mathbb {Q}}(t)+\sqrt{(1-\rho _i^2)}dZ_i^{\mathbb {Q}}(t)\right) \nonumber \\ \end{aligned}$$

Given these differential equations, the evolution of the forward rate is defined based on 2*N* standard Wiener processes ($W_i^{\mathbb {Q}}(t)$,$Z_i^{\mathbb {Q}}(t)$). *N* defines the number of dimensions of the model. In Eq. (2), $\mu _f(t,T)$ equals the forward drift. Under the assumption of no-arbitrage, Heath et al. (1992) have shown that this term is defined as:

4 $$\begin{aligned} \mu _f(t,T)=\sum _{i=1}^{N}v_i(t)\sigma _{f,i}(t,T)\int _{t}^{T}\sigma _{f,i}(t,u)du \end{aligned}$$

Based on this property, the evolution of the forward rate under the risk-neutral measure is solely driven by the initial forward rate curve, the volatility state variables ($v_i(t)$) and the volatility function ($\sigma _{f,i}$). Within the TS model, the volatility function is set to a specific form (see Eq. 5) to ensure that the forward rate can be represented by a finite-dimensional Markov process and a time-homogeneous volatility structure as:

5 $$\begin{aligned} \sigma _{f,i}(t,T) = \left( \alpha _{0,i}+\alpha _{1,i}(T-t)\right) \cdot e^{-\gamma _i(T-t)} \end{aligned}$$

The TS model offers semi-analytical pricing for the most common interest rate products. In this paper, we use swaptions prices as input for the calibration of the TS model, in line with the benchmark implementation. Hence, we need to calculate the prices of swaptions under the TS model. The TS model provides a semi-analytical solution for an option on a zero-coupon bond. We perform the pricing of swaptions by utilizing these pricing functions and mapping the swaptions based on the stochastic duration method (Munk 1999).^4^

The TS model is applied in the given benchmark implementation and considered to be suitable to assess the performance of the calibration framework. Furthermore, the TS model offers a semi-analytical solution for pricing European Swaptions, which will be used as calibration instruments for our empirical study. Hence, we are able to generate train and test data in a fast and efficient way. Nevertheless, the model is complex enough to capture the structure and properties of the market-implied volatility/price cube. The TS model can be transformed into more simplistic IR term structure models by simply using specific settings for the parameters of the volatility function (see Trolle and Schwartz 2009). Hence, our results are also relevant for the application of ANNs to calibrate more simplistic IR term structure models, which are also common in practical implementations.

**Table 1:** Parameters of the Trolle–Schwartz model

Parameter	Interpretation
$\kappa$	Mean reversion speed of the variance process
$\theta$	Long-term variance
$\sigma$	Volatility of the variance
$\rho$	Correlation between forward rate and volatility state variables
$\alpha _{0}$	Free parameter of the volatility function $\sigma _{f}(t,T)$
$\alpha _{1}$	Free parameter of the volatility function $\sigma _{f}(t,T)$
$\gamma$	Free parameter of the volatility function $\sigma _{f}(t,T)$

This table provides an overview of the model parameters in the TS model and their interpretation

As discussed above, the calibration of a model requires the setting of model parameters such that the model prices fit the observable market prices. The calibration of the TS model requires the determination of *N*x7 parameters (see Table 1). We consider these parameters as elements of *N* parameter vectors $\Omega _i$. In line with the setup of the benchmark implementation, we set $N=1$ which reduces the calibration problem to the determination of seven parameters.^5^ In our empirical study, we perform a daily calibration of these parameters by using the sum of squared errors over a set of observable swaption prices as loss function. Hence, the specific calibration procedure for the TS model can be written as:

6 $$\begin{aligned} {{\,\mathrm{arg\,min}\,}}_{\Omega _t} \sum _{j\in \mathscr {F}_t} \left( p_j^{(market)}-\hat{p}_j^{(model)}\left( \Omega _t \ |\ \tau _j,\Lambda _t\right) \right) ^2, \end{aligned}$$

where $\Omega _t$ equals the parameter vector ($\Omega _t=(\kappa _t,\theta _t, \sigma _t,\rho _t,\alpha _{t0}, \alpha _{t1},\gamma _t)$) for a specific trading day (*t*). In case of IR swaptions, $\tau _j$ equals a vector of properties describing the instrument, such as expiry date of the swaption, tenor and swap rate of the underlying swap. $\Lambda _t$ represents the yield curve (and discount factors) in the respective currency. Based on these inputs a model price is calculated. The calibration procedure optimizes $\Omega _t$ such that the loss function is minimized. The number of available instruments in the empirical application is much higher than the number of parameters to calibrate in the TS Model $(\mathscr {F}_t> \Omega _t )$. Therefore, we do not add an additional penalty term in Eq. (6) to counteract overfitting, in contrast to the original CaNN framework of Liu et al. (2019).^6^ The swaptions used in the empirical section are consistent with the price observations entering the calibration in the benchmark implementation. This means, that we only use swaptions that are sufficiently liquid. Furthermore, we do not introduce a weighting function in Eq. (6) to focus on the calibration of ATM swaptions, which is in line with the calibration setting at the financial institution. We refer to $\Omega _t^{BM}$ for the values calibrated by the benchmark implementation at the financial institution and to $\Omega _t^{ANN}$ for the calibrated values of our approach. The observable market prices are structured along three dimensions (expiry tenor, swap tenor, strike). Hence, the observable swaption data can be thought of as a cube of swaption prices.

## ANN calibration approach

### Methodological overview

**Fig. 1:**
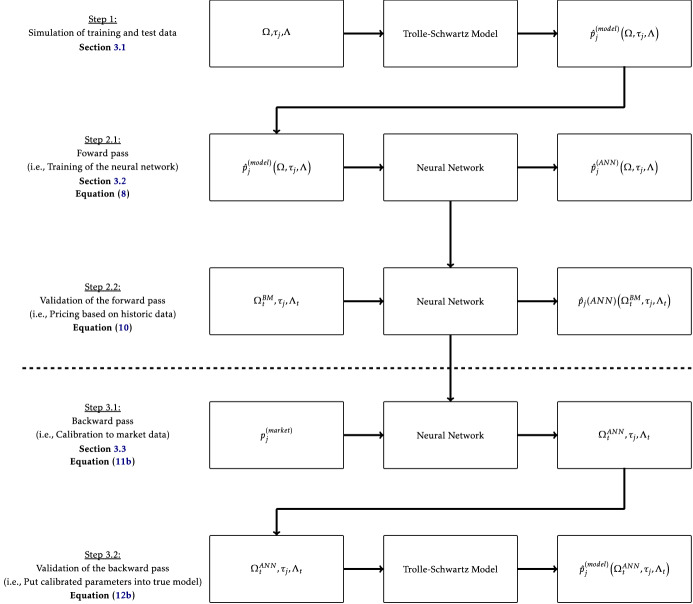
Workflow of the CaNN framework. Note: This figure is a detailed description of the calibration framework (CaNN). In the first step, we simulate millions of swaptions based on the Trolle-Schwartz model. In step 2.1, we train the neural network such that the sum of squared differences between the model prices and the ANN prices is as small as possible. Step 2.2 is an important validation step. We put the real historic values of $\Omega ^{BM}_t$, which are calibrated by the benchmark implementation of the financial institution, into the trained neural network and compare the squared difference between the $\hat{p}_j^{(model)}\left( \Omega _{t}^{BM}, \tau _j,\Lambda _t\right)$ and $\hat{p}_j^{(ANN)}\left( \Omega _{t}^{BM}, \tau _j,\Lambda _t\right)$. The smaller the value, the better our ANN approximates the semi-analytical pricing function used in the benchmark implementation. In step 3.1 we put the observed market prices of each trading day into the neural network and try to find the values of $\Omega _t^{ANN}$ which produces the smallest deviations of $\hat{p}_j^{(ANN)}\left( \Omega _{t}^{ANN}, \tau _j,\Lambda _t\right)$ and $p_j^{(market)}$ for all observable swaptions for a given trading day. To ensure that the parameter combination $\Omega _t^{ANN}$ is also a valid solution in the true model, we put the values $\Omega _t^{ANN}$ into the Trolle-Schwartz model in step 3.2 and compare the differences between $\hat{p}_j^{(model)}\left( \Omega _{t}^{ANN}, \tau _j,\Lambda _t\right)$ and $p_j^{(market)}$. In each of these steps we want to achieve a similar level of accuracy compared to the given benchmark implementation of the financial institution, as the advantages of the CaNN framework are speed and less computational resources providing similar calibration errors.

In general, a calibration framework should be flexible, robust, fast and accurate. All these properties are combined in ANNs. They became widespread in the financial domain due to their flexibility and approximation properties. We use the calibration framework (CaNN) proposed by Liu et al. (2019), which involves two consecutive components (two-step or indirect approach). First, we train an ANN to learn the pricing functions for swaptions under the TS model (forward pass). Second, the resulting ANN is applied within a calibration procedure, to fit the model parameters ($\Omega$) to a set of observable market prices. There are other publications that suggest a one-step (direct) approach, where model parameters are learned from market prices directly (e.g. Gambara and Teichmann 2020 or Hernandez 2017).^7^ The indirect approach has a series of advantages compared to the direct approach when it comes to the practical application of ANNs for calibration of financial models (see Horvath et al. 2021 and Bayer et al. 2019 for a comprehensive discussion of reasons for preferring the two-step approach). Most importantly, the two-step approach leverages on existing knowledge and experiences with respect to traditional pricing models and leads to a deterministic calibration framework (Horvath et al. 2021). These aspects could ease the discussion with regulators, when introducing the prevailing calibration framework in practice. Furthermore, the separation of the pricing and calibration procedure makes it easier to explain results and identify sources of deviations from market prices. Based on this discussion, we prefer an indirect (two-step) approach for the practical application of the ANN calibration framework. Figure 1 illustrates the subsequent steps of the calibration framework, which are outlined in the rest of this section.

ANNs are capable of approximating any continuous function that maps input variables to outputs, see Cybenko (1989) and Hornik (1991). Our approach utilizes this principle to map input features on swaption prices in a highly non-linear and complex fashion. For each swaption, the neural network starts with covariates $(\Omega ,\tau _j,\Lambda ) \in \mathbb {R}^{p}$ as inputs which are called input neurons. The network consists of stacked layers $l = 1,\ldots , L$ whereby each layer consists of $k_l = 1,\ldots , K_l$ neurons $\varvec{h}_{l_{K_l}} \in \mathbb {R}^{K_l}$ that are determined by an affine combination of neurons in the previous layer which is composed with an arbitrary (non-linear) activation function $\delta$. Formally, the ANN is defined by:^8^

$$\begin{aligned} \varvec{h}_{l_{K_l}} = \delta \left( \varvec{W}_l \varvec{h}_{(l-1)_{K_{l-1}}} + \varvec{b}_l \right) \end{aligned}$$

with $\varvec{W}_l \in \mathbb {R}^{K_l \times K_{l-1}}, \varvec{b} \in \mathbb {R}^{K_l}$ as parameters which are usually called weights and biases. Estimates are derived from the last layer, the so called output layer and are given by choosing the identity function for $\delta$, resulting in:

$$\begin{aligned} F \left( y | \varvec{x} \right) = \varvec{W}_{L+1} \varvec{h}_{K_L} + \varvec{b}_{L + 1} \end{aligned}$$

### The forward pass: learning the pricing function

Step 1 is a prerequisite for the approximation of the TS model using the ANN. In this step, we generate millions of different swaptions to train the ANN. However, this is the most computationally intense part of the whole setup. A detailed description of the swaption characteristics and the range of parameters can be found in Sect. 4.1. Step 2.1 of the calibration framework consists of learning the mapping function, i.e. the Trolle-Schwarz Model, via an Artificial Neural Network (ANN). Finding a suitable architecture which holds the balance between computational time, complexity and accuracy is the main task in this subsection. As our goal is a highly accurate approximation, we use a rather large and complex neural network, as it ensures a high approximation accuracy. As ANNs are sensitive to diverging dimensions of input parameters, we normalize all features $\xi \in (\Omega ,\tau _j,\Lambda )$ to a predefined range, i.e $\xi \in [\xi _{min},\xi _{max}]$, closely following Horvath et al. (2021). This makes it also easier in the backward pass to set optimization bounds. The features are normalized by:

7 $$\begin{aligned} \frac{2\xi -(\xi _{max}+\xi _{min})}{\xi _{max}-\xi _{min}}\in [-3,3]. \end{aligned}$$

Usually, ANNs are prone to the problem of overfitting, meaning, that the network is able to approximate the training data very well, but fails to approximate unseen test data. This is usually the case in out-of-time prediction in the financial context. Our approach is not designed to provide a prediction in an out-of-time fashion, as we want to approximate a specific mapping function as accurate as possible. In our case, the mapping function of training and test data is equal, as both datasets are generated via the (highly complex) pricing function for swaptions under the TS model. As stated by Srivastava et al. (2014), some of these relationships will occur only due to sample noise, resulting in overfitting complex relations in the training set. This could be averted by increasing the number of observations. As we use simulated data for the training of the ANN, we can ensure a large sample size. Furthermore, the data generating process we want to approximate has no inherit noise, as the relation between input parameters and the resulting prices in the TS model is deterministic. Therefore, the ANN is not prone to the problem of overfitting the noise of the data.

Furthermore, in the empirical section, the number of simulated swaptions is larger than the parameters to be estimated by the neural network. Hence, this optimization is overdetermined, which also reduces the chance of overfitting, see Bishop (2006).^9^ Therefore, we are confident that approximating the training data ensures that the test data is approximated similarly well. Hence, the issue of overfitting can be neglected in the prevailing use case, as shown by our empirical results in Sect. 4.2. Furthermore, this is supported by findings of previous papers, such as Liu et al. (2019) and Liu et al. (2019). These authors conduct hyper parameter searches, including techniques to reduce overfitting. In none of their final models, an overfitting reducing technique is found to be beneficial for the quality of the ANN’s approximation. Hence, these findings underline the above mention indications that the problem of overfitting can be neglected when learning the mapping function within an ANN based calibration framework. Of course, this only holds if we generate a vast amount of training data, which can easily be ensured here. For a detailed description of the generation of the training data, we refer to Sect. 4.1.

The ANN is trained to minimize the following loss function^10^ with respect to weights $\varvec{W}$ and biases $\varvec{b}$:

8 $$\begin{aligned} {{\,\mathrm{arg\,min}\,}}_{\varvec{W}, \varvec{ b}} \sum \left( p_j^{(model)}(\Omega ,\tau _j,\Lambda )-\hat{p}_j^{(ANN)}(\varvec{W}, \varvec{ b}\ |\ \Omega ,\tau _j,\Lambda )\right) ^2 \end{aligned}$$

As a precaution, we also generated test samples to calculate the loss of Eq. (8) in an out-of-sample task. In general, the ANN is trained over 5000 epochs to ensure the weights and biases are estimated as accurate as possible. In the additional validation step 2.2, we test the approximation properties of the ANN on real market data. We use the historical parameter values calibrated by the financial institution, put them into the ANN and compare the resulting prices with the observed market prices. We do this for a time period not included in the training of the ANN, i.e. parameter values and yield curves are unseen to the ANN. This step should give a first indication of robustness to unseen market periods.

### The backward pass: calibration of model parameters

Step 3.1 of the framework is to calibrate the input parameters $\Omega _t$ given the observed market prices at a specific trading day (*t*). After the forward pass is successfully accomplished, the weights and biases describing the relation of the input parameters $(\Omega _t,\tau _j,\Lambda _t)$ to the prices of a swaption $p_j$ are known. This means that the mapping function is now deterministic in the sense that simple and fast matrix multiplications map the input to the corresponding swaption prices ($\hat{p}_j^{(ANN)}$). Hence, we have now a very fast way to price a swaption given $(\Omega _t,\tau _j,\Lambda _t)$. For calibration purposes, we are interested in $\Omega _t$ which expresses the observed market prices $p_j^{(market)}$ based on the TS model as good as possible. Hence, we basically invert the trained neural network by setting the values of $\Omega _t$ as degrees of freedom in a optimization problem:

9 $$\begin{aligned} {{\,\mathrm{arg\,min}\,}}_{\Omega _t} \sum _{j\in \mathscr {F}_t}\left( p_j^{(market)} -\hat{p}_j^{(ANN)}(\Omega _t\ |\ \tau _j,\Lambda _t, \varvec{W}, \varvec{ b})\right) ^2 \end{aligned}$$

The optimization problem in Eq. (9) is essentially the calibration problem widely faced in the financial industry. To solve this problem, usually local optimizers are widely used due to their speed (see Liu et al. 2019 or Gambara and Teichmann (2020)). In our analysis, several local minima exist, see e.g., Gilli and Schumann (2012). This may be a bottleneck for local optimizers. As we gain a high amount of speed by using the neural network approach, we are able to use slower, but in terms of minimization more robust optimizers. In the calibration framework, we apply a global optimizer called differential evolution (see Storn and Price 1997 for more details).^11^ This stochastic optimization scheme is probably able to find a global minimum even if the optimization problem is non-convex. We speed up the calibration framework by using the (transformed) values of $\Omega _{t-1}$ as initial values for the optimization (this is also done by the benchmark implementation).

## Empirical study

### Data

**Fig. 2:**
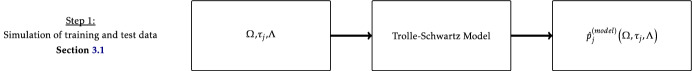
The CaNN framework | Simulation of training and test data. Note: In the first step, we simulate millions of swaptions based on the Trolle-Schwartz model.

The first step of the calibration framework is to simulate millions of different swaptions to train the ANN. This is a computationally intensive step, but has to be done only once. Figure 2 illustrates this initial step of the calibration framework.

Our empirical study is based on a comprehensive set of daily prices for EUR swaptions. These prices are used as input for the calibration procedure. The available market data covers 439 consecutive trading days from January 2019 to September 2020. Hence, our dataset includes the stressed market period in the context of the COVID-19 pandemic in spring 2020. The daily swaption data is available for different expiry tenor, swap tenor and strike values:

- Option Tenor: 1M, 3M, 6M, 9M, 1Y, 2Y, 5Y, 10Y, 15Y, 20Y
- Swap Tenor: 1Y, 2Y, 5Y, 10Y, 15Y, 20Y, 30Y
- Strike (ATM ± bp): 0, 12.5, 25, 50, 100, 150, 200

On each trading day, we observe valid prices for about 800 swaptions. This amounts to a total number of more than 350,000 price observations. In practical applications, financial institutions tend to use a reduced set of swaptions for the calibration of IR term structure models to reduce the calibration time. For our empirical study, we do not further reduce the amount of swaptions entering the calibration procedure to be in line with the benchmark implementation. In addition to swaption data, we obtain the yield curve (6m EURIBOR) for each trading day as well as the relevant forward rate for each swaption. The yield curve is transformed into discount factors for 53 tenors. We compare our calibration performance against the benchmark implementation, which is using a Levenberg–Marquardt optimization algorithm (see Levenberg 1944; Marquardt 1963) by iterating the traditional pricing formula using a large computing cluster using 72 CPU cores simultaneously. In contrast, the ANN calibration procedure is based on a standard office computer with 8 CPU cores used at the same time.^12^

The data for each trading day includes the model parameters and model prices estimated by the benchmark implementation. Table 2 provides an overview of the observed values for each TS parameter and the associated model prices.

**Table 2:** Training and market data

Parameter	Observed (Benchmark)	Sampling (CaNN)
Kappa ($\kappa$)	[0.0031,2.80]	[0.005,3]
Theta ($\theta$)	[0.037,3.89]	[0.01,4.0]
Sigma ($\sigma$)	[0.24,1.73]	[0.1,2.0]
Rho ($\rho$)	[$-0.047$,0.60]	[$-0.50$,0.80]
Alpha0	[0.00001,0.006]	[0.00001,0.008]
Alpha1	[0.0007,0.005]	[0.0005,0.005]
Gamma ($\gamma$)	[0.048,0.089]	[0.01,0.1]
Prices $\left( \hat{p}_i^{(model) }\right)$	[0.0,0.64]	[0.0,1.06]

This table provides observed values for Trolle–Schwartz parameters as well as the value ranges used for sampling of training data

As discussed in Sect. 3.1, we do not perform the training with real swaption market data. While our swaption dataset includes 350,000 observations, it only provides 439 combinations of TS model parameters. Hence, the number of observations is not sufficient to ensure a satisfying performance of the ANN.

For Step 1 in Fig. 1, i.e. to train the ANN, we need to generate a large amount of artificial (synthetic) swaption data. We get the required dataset by sampling swaption data for 12,000 synthetic trading days. By using synthetic swaption data for training and testing, we are able to set aside the swaption prices obtained from real market data for the validation of the ANN. The properties of the synthetic swaptions are set to the discrete values shown above. The values for the TS model parameters are randomly sampled from predefined ranges (see Table 2) using a uniform distribution. Please note that in general the value ranges used for sampling of parameter values exceed the observed parameter values of the benchmark implementation. Thereby, we ensure that the calibration procedure is able to provide prices for parameter values outside of observed ranges. Furthermore, the CaNN framework is able to find optimal parameter values outside the observed ranges in the calibration procedure.

The yield curve for each synthetic trading day is randomly sampled from a collection of yield curve data. The yield curve dataset is constructed by a blended approach, where we combine historically observed market data with synthetic yield curve data. First, we collect yield curves for eight different currencies^13^ for a historic two-year time period (Apr 2018–Apr 2020). This includes about 3700 different yield curves. We do not include the yield curves observed from May until September 2020 to obtain a real out-of-time validation of the CaNN calibration results within our empirical analysis. Second, we enrich the dataset by adding 20,000 synthetic yield curves. These yield curves are generated by using an algorithm based on the Nelson-Siegel-Svensson methodology (see Nelson and Siegel 1987; Svensson 1994). Our blended approach provides a comprehensive and representative yield curve dataset. On the one hand, we consider recent historic market environment in the training process. On the other hand, we ensure that the resulting ANN is flexible enough to cope with new unseen market data. Furthermore, this approach offers the possibility for recurring generation of training data and re-training of the CaNN framework based on newly observed yield curves.

By following the generation procedure outline above, we obtain a total number of 9.6 million synthetic swaptions. The prices of these swaptions are calculated by applying the pricing procedure outlined in Sect. 2.3. The resulting dataset is used for training and testing the ANN in Step 2.1, see Fig. 1, of the calibration framework. In general, we consider the generation of training and test data as a crucial and probably the most laborious task within the calibration framework. The composition of the dataset and its granularity are important drivers of the CaNN’s estimation power. Please note that the initial training of the ANN is time consuming and requires significant computational capacities. Nevertheless, this step has to be performed only once. The application of the CaNN framework can be accompanied by frequent re-training, which is significantly less time consuming.

### ANN architecture and forward pass (pricing)

**Fig. 3:**
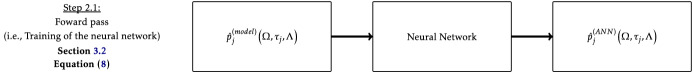
The CaNN framework | The forward pass. Note: In step 2.1, we train the neural network such that the sum of squared differences between the model prices and the ANN prices is as small as possible.

After simulating millions of swaptions, the training of the ANN is the subsequent step. Hereafter, we optimize the network architecture and determine the weights and biases to approximate the TS model as close as possible. Figure 3 provides a graphical representation for this step of the calibration framework.

Finding a suitable ANN architecture is a major cornerstone of the successful approximation of the pricing function. As usual, one has to find the balance between approximation accuracy and computational burden, hence a so called random search of the hyper parameters with a subset of the training data is employed. Resulting from this, four hidden layers with 2048, 1024, 512 and 256 neurons are used. To optimally train the ANN, we use the *Adam* optimizer and Relu activation function. As described above, we do not use any dropout layer or early stopping criterion. To ensure convergence with the TS model, we train the ANN with 5000 epochs. An overview of the hyper parameters is illustrated in Table 3.

**Table 3:** Hyper parameter of the CaNN

Parameter	Value
Number Features (X)	66
Hidden Layers	4
Neurons per Layer	[66, 2048, 1024, 512, 256, 1]
Number of parameters	2,891,777
Loss function	Sum of squared errors
Activation function	ReLu
Optimizer	Adam
Initialization	Glorot-Uniform
Batch Size	16,384

This table provides the applied hyper parameters of the final CaNN. In total, a neural network with four hidden layers and 2,891,777 parameters is trained to approximate swaption prices under the TS model

For illustration, we also employed and validated the hyper parameter setting proposed by Liu et al. (2019) with 200 neurons in each of the four hidden layers. The accuracy in terms of mean squared error is 10 times worse than with our architecture. This gives rise to the conjecture that any calibration framework needs a tailored set of hyper parameters to provide the a sufficiently accurate estimation of model prices. This also suggests, that the model complexity of the ANN should increase with the complexity of the IR dynamics.^14^ To train the ANN, we randomly split the 12,000 synthetic trading days into a training set (7.68 million swaptions) and a test set (1.92 million swaptions). Table 4 shows key evaluation metrics in the train and test sample.

**Table 4:** Results of ANN training

CaNN	MSE	MAE	RMSE
Training	1.47e−07	2.38e−04	3.52e−04
Testing	1.80e−07	2.45e−04	4.24e−04

This table provides the performance measures for the ANN training. As all six measures are quite low, we are confident that the ANN approximates the TS model very well

**Fig. 4:**
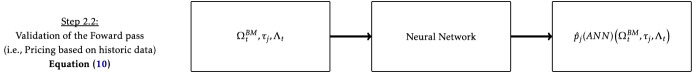
The CaNN framework | Validation of the forward pass. Note: Step 2.2 is an important validation step. We put the historic values of $\Omega ^{BM}_t$, which are calibrated by the benchmark implementation into the trained neural network and compare the squared difference between the $\hat{p}_j^{(model)}\left( \Omega _{t}^{BM}, \tau _j,\Lambda _t\right)$ and $\hat{p}_j^{(ANN)}\left( \Omega _{t}^{BM}, \tau _j,\Lambda _t\right)$. The smaller the value, the better our ANN approximates the semi-analytical pricing function used in the benchmark implementation.

We observe only small differences, when comparing the results for the train and test set. This may imply that the ANN generalizes well and we do not encounter overfitting. Furthermore, the metrices are well in line with results of previous studies, see e.g. Liu et al. (2019) or Horvath et al. (2021). The very similar performance for the train and test data may also be attributed to the comparatively large training sample, which is imminent to approximate the mapping function accurately. After training the neural network, we validate our results against an implementation of a large financial institution in step 2.2, see Fig. 4:

In contrast to most other papers on the application of ANNs for pricing and calibration, we perform an additional validation of the forward pass based on historic pricing data obtained from a benchmark implementation (BM). We call this step the “out-of-simulation validation“, as the data used to assess the ANN’s pricing performance has not been generated with the same process as the train and test sample, but historically based on real-life market data. Thereby, we ensure that the ANN has learned the TS pricing function correctly and performs well in a true out-of-sample evaluation. From our point of view, the validation based on results from a benchmark model is a prerequisite for the practical application of an ANN based calibration framework. To perform the out-of-simulation validation, we pass the observed parameters estimated by the benchmark implementation ($\Omega ^{(BM)}$) together with the historic market data for the respective trading day through the ANN for all swaptions across available trading days. Afterwards, we compare the predicted prices of the trained ANN with the model prices generated by the benchmark implementation (see Eq. 10 for mathematical illustration).

10 $$\begin{aligned} MSE=\frac{1}{T}\sum _{t=1}^{T} \sum _{j\in \mathscr {F}_t} \left( \hat{p}_j^{(model)}\left( \Omega _t^{(BM)}\ |\ \tau _j,\Lambda _t\right) -\hat{p}_j^{(ANN)}\left( \Omega _t^{(BM)}\ |\ \tau _j,\Lambda _t, \varvec{W}, \varvec{ b}\right) \right) ^2 \end{aligned}$$

The results of this validation step are displayed in Table 5. First, we check the performance for the time period from January 2019 to April 2020. The swaption data from this period was used for setting the parameter ranges and yield curves for the simulation of synthetic swaptions. As the evaluation metrics are close to the results obtained in the training and testing, we may conclude that the ANN is robust in real-life market situations. As a next step, we use the benchmark parameters from the out-of-time period (May 2020–September 2020). Data and information from this period, such as parameter values and yield curves, have not been used in the previous steps and is therefore completely new to the framework. The results for this period of time indicate that we achieved generalization even in an out-of-time perspective with unseen circumstances. These results may serve as a first proof of concept for a practical implementation.

**Table 5:** Results of ANN training

CaNN	MSE	MAE	RMSE
Out-of-simulation (Jan 2019–Apr 2020)	5.47e−07	2.98e−04	7.24e−04
Out-of-simulation (May 2020–Sept 2020)	2.48e−07	2.65e−04	4.98e−04

This table show key evaluation metrics in the out-of-simulation validation. We divide the samples into data building the basis of our training (January 2019 to April 2020) and true out-of-time data (May to September 2020)

**Fig. 5:**
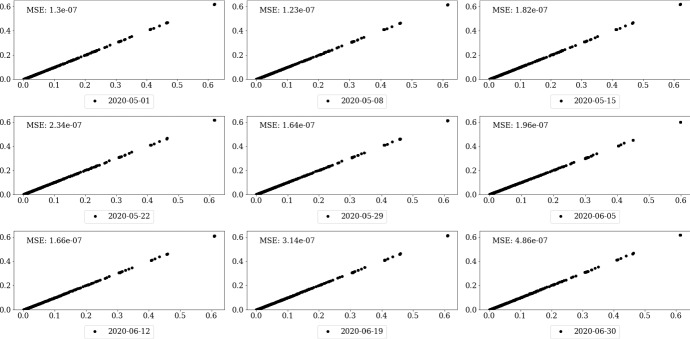
Real fit plots for selected trading days. Note: These figures show the real fit plots of selected historic trading days. Furthermore, the day specific MSE is displayed. The price estimations of the ANN are displayed on the x-axis, whereas the model prices of the benchmark implementation is shown on the y-axis.

Figure 5 provides real fit plots for selected trading days taken from the out-of-time period. The plots compare the prices estimated by the ANN (x-axis) with model prices from the benchmark implementation (y-axis). As we can see, the points are on the bisecting line which implies a very good convergence of the ANN prices to BM model prices. To each real fit plot, the MSE for the respective trading day is added. For some days, we obtain much better results than in training, whereas for other days we are slightly worse. In summary, we find sufficient evidence that the trained ANN generalizes very well even if confronted with unseen data. Hence, the ANN provides a very good approximation of the TS pricing function for swaptions.

### The backward pass (calibration)

**Fig. 6:**
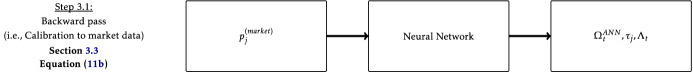
The CaNN framework | The backward pass. Note: In step 3.1 we put the observed market prices of each trading day into the neural network and try to find the values of $\Omega _t^{ANN}$ which produces the smallest deviations of $\hat{p}_j^{(ANN)}\left( \Omega _{t}^{ANN}, \tau _j,\Lambda _t\right)$ and $p_j^{(market)}$ for all observable swaptions for a given trading day.

For the rest of the section, we are now concerned with the calibration task frequently performed by the given benchmark implementation. For step 3.1 of the calibration framework, we utilize the trained ANN to calibrate the TS model parameters to a daily set of observable swaption prices, see Fig. 6. Furthermore, we validate our results against the real-life benchmark implementation of a large financial institution.

For each of the 439 trading days, we obtain two calibrated parameter sets. One parameter set is returned from the benchmark implementation ($\Omega _t^{(BM)}$), while the other parameter set results from the ANN based calibration framework ($\Omega _t^{(ANN)}$). For clarification, we restate and concretize the general formulation of the calibration problem in Eq. (9) and provide a specific notation for both calibration processes:

11a $$\begin{aligned}&{{\,\mathrm{arg\,min}\,}}_{\Omega _t^{(BM)}} \sum _{j\in \mathscr {F}_t}\left( p_j^{(market)} -\hat{p}_j^{(model)}(\Omega _t^{(BM)}\ |\ \tau _j,\Lambda _t)\right) ^2 \end{aligned}$$

11b $$\begin{aligned}&{{\,\mathrm{arg\,min}\,}}_{\Omega _t^{(ANN)}} \sum _{j\in \mathscr {F}_t}\left( p_j^{(market)} -\hat{p}_j^{(ANN)}(\Omega _t^{(ANN)}\ |\ \tau _j,\Lambda _t, \varvec{W}, \varvec{ b})\right) ^2 \end{aligned}$$

Both calibration approaches aim to minimize the sum of squared errors for each trading day. By minimizing the loss function, an optimal set of TS model parameters is selected. The benchmark implementation performs the calibration by applying a local optimization algorithm (Levenberg–Marquardt) and repeatedly calls the traditional implementation of the semi-analytic pricing formula (see Eq. 11a) and sets parameter restrictions for the TS parameters to ensure that the optimizer returns a result. For this empirical analysis, the benchmark model parameters ($\Omega _t^{(BM)}$) are obtained from the historical calibration results of the benchmark implementation. The CaNN framework utilizes the forward pass by frequently estimating swaption prices based on the trained neural network for different parameter settings (see Eq. 11b). Please note that the weights and biases of the ANN have already been set in the training phase (forward pass) and are not altered during the calibration procedure.

With respect to the substantial acceleration using the ANN, a global optimization algorithm (differential evolution) can be used to minimize the loss function given by Eq. (11b). Due to time constraints in the productive workflow of the financial institution, only a local optimizer is used in the benchmark setup. The application of the differential evolution (DE) algorithm shall avoid the problem of stopping at local minima and offers the advantage that no starting values are required (see Liu et al. 2019). However, we use the parameter values of the previous trading day as starting values for the DE algorithm. We observe that using starting values leads to a faster convergence and significantly accelerates the calibration process. In practical applications, such as the referred benchmark implementation, the parameter values of the previous trading day are commonly used as starting point for the optimization process. This could potentially lead to a deterioration of the minimization, when applying local optimizers, but should not be an issue for global optimization algorithms. Hence, we are confident that there is no downside in setting starting values for the DE algorithm in the CaNN framework. On the contrary, we observed that setting starting values speeds up the ANN calibration by roughly 50 times. Thereby, the calibration for each trading day can be performed in about 30 s. This is roughly four times faster than the benchmark implementation, although it uses a local optimizer and 72 CPU cores. This means, that our approach, i.e. using a global optimizer and only 8 CPU cores, is faster than the benchmark implementation. Summarizing, we can achieve a very similar calibration error, see Table 6, but are faster, require less computational resources and are able to use a global optimizer. Even more benefits could be realized if the financial institutions use financial models without analytical solutions, i.e. the prices can only be determined via Monte Carlo simulations. However, this would increase the computational burden of the first step greatly, as the generation of enough training data could take extremely long.

**Table 6:** Calibration results

Period	Daily MSE (BM)	Daily MSE (ANN)	Daily SSE (BM)	Daily SSE (ANN)
Jan 2019–Apr 2020	1.36e−06	1.29e−06	1.11e−03	1.10e−03
May 2020–Sept 2020	1.61e−06	1.63e−06	1.13e−03	1.13e−03

This table show key evaluation metrics of the ANN and benchmark calibration result. We divide the samples into data building the basis of our training (January 2019 to April 2020) and true out-of-time data (May to September 2020)

Table 6 provides an overview of the calibration results equal to the average daily values of the loss function calculated by Eqs. (11a) and (11b) as well as the daily mean squared error (MSE) for both calibration approaches. The results show that the CaNN framework provides calibration results that are very close to the benchmark implementation for both time periods.

Nevertheless, there might be a concern that these results do not provide sufficient evidence for the practical applicability of the CaNN framework. We expect that supervisory authorities will have a critical view on the application of ANNs for pricing and calibration as the ANN pricing function constructed in the forward pass is not considered traceable given the high amount of parameters in the neural network.

**Fig. 7:**
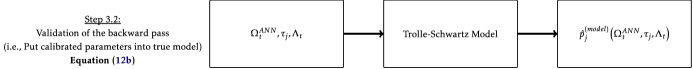
The CaNN framework | Validation of the backward pass. Note: To ensure that the parameter combination $\Omega _t^{ANN}$ is also a valid solution in the true Trolle-Schwartz model, we put in the values $\Omega _t^{ANN}$ into the Trolle-Schwartz model in step 3.2 and compare the differences between $\hat{p}_j^{(model)}\left( \Omega _{t}^{ANN}, \tau _j,\Lambda _t\right)$ and $p_j^{(market)}$.

To prove that the CaNN provides reliable parameter values, the calibration framework involves an additional validation step 3.2. Hence, the CaNN parameter set ($\Omega _t^{(ANN)}$) is used as input for the semi-analytical pricing formula for swaptions under the TS model. By comparing the resulting prices with observable market prices, we are able to prove that the CaNN calibration results hold true in the Trolle–Schwartz model framework, see Fig. 7:

Hence, we apply Eq. (12b) to validate the ANN solution for each trading day. The result will provide insights with respect to the true quality of the CaNN calibration results.

12a $$\begin{aligned} SSE^{(BM)}(t)= & {} \sum _{j\in \mathscr {F}_t}\left( p_j^{(market)} -\hat{p}_j^{(model)}(\Omega _t^{(BM)}\ |\ \tau _j,\Lambda _t)\right) ^2 \end{aligned}$$

12b $$\begin{aligned} SSE^{(ANN)}(t)= & {} \sum _{j\in \mathscr {F}_t}\left( p_j^{(market)} -\hat{p}_j^{(model)}(\Omega _t^{(ANN)}\ |\ \tau _j,\Lambda _t)\right) ^2 \end{aligned}$$

**Fig. 8:**
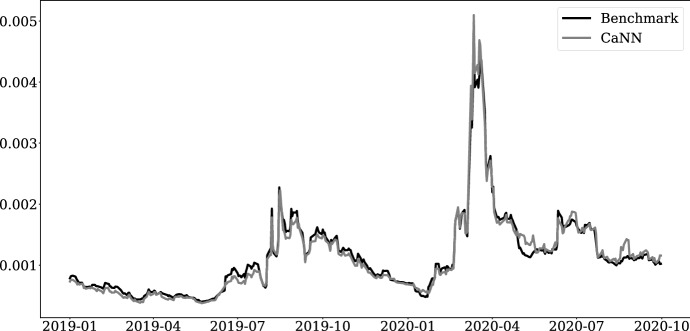
Sum of squared errors over trading days. Note: This figure shows the sum of squared errors of trading days for the whole time span. The grey line corresponds to the SSE using the CaNN approach, whereas the black line coincides with the SSE of the benchmark implementation.

Figure 8 illustrates the daily performance measure (SSE) for both calibration approaches over time. The black line represents the benchmark result (Eq.  12a), while the grey line represents the performance measure for the CaNN framework (Eq. 12b). In general, we find that the performance of both calibration approaches significantly varies over time. In the early months of 2019 the losses are comparatively low whereas in the fourth quarter of 2019, we observe a considerable increase. A remarkable spike can be observed after the break-out of the COVID-19 pandemic, meaning that the calibrated TS model prices strongly deviates from market prices. These results clearly indicate that a thorough assessment of ANN calibration approaches should be done in different market environments to ensure their practical applicability.

The results presented in Fig. 8 show that the CaNN framework produces competitive results compared to the benchmark implementation in terms of daily performance. For some market periods we can even find better solution for the parameters, see e.g. the period from June 2019 to August 2019 or the early months of 2019. The largest deviation between the CaNN and the benchmark implementation can be observed during the COVID-19 period in the March 2020. Nevertheless, the daily performance of both approaches does not differ significantly even in this stressed market environment. Hence, the CaNN framework does provide comparable calibration results even in extreme and unusual market situations in a faster and computationally more efficient manner. Furthermore, the very good results for the out-of-time period (May to September 2020) indicate that the performance of the CaNN framework does not depend on including current market data during training.

**Fig. 9:**
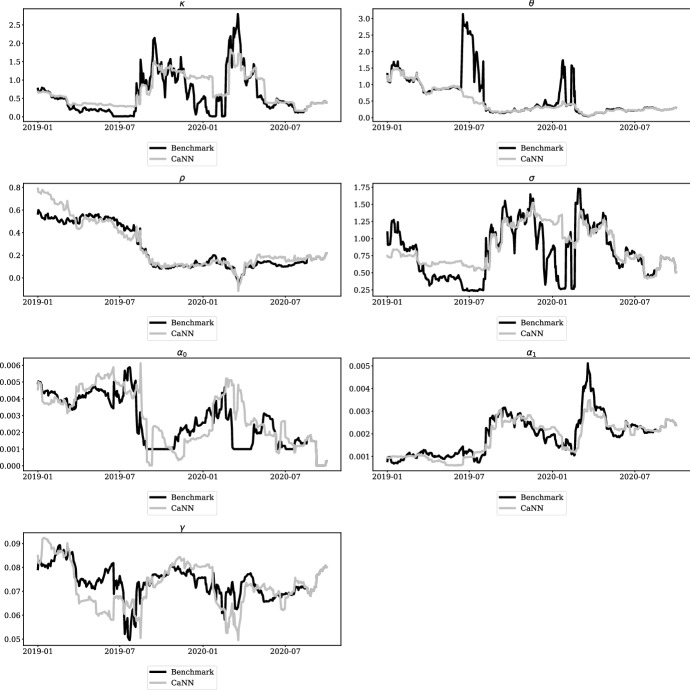
Calibrated parameters over trading days. Note: These figures show the calibrated values of $\Omega _t^{(BM)}$ and $\Omega _t^{(ANN)}$. The black line represents the values gathered form the benchmark implementation, whereas the grey line illustrates $\Omega _t^{(ANN)}$. For details on the parameters, please refer to section 2.

In addition to analyzing the performance of the CaNN framework, we are interested in a comparison of the parameter estimates for both calibration approaches. Figure 9 illustrates the different estimates for all elements of $\Omega _t$ over time. The black line represents the parameter estimated by the benchmark implementation, while the grey line represents the respective element of $\Omega _t^{(ANN)}$.

Overall, the analysis reveals that parameter estimates from both calibration procedures are quite close to each other and have a similar evolution over time. However, the results indicate that the CaNN parameters are more stable over time and therefore more robust against taking extreme values.^15^ For example the BM estimates for $\theta$ show four considerable peaks in the analyzed period, while the CaNN estimates show a relatively smooth evolution over time. On some days, the benchmark implementation obtains extreme values for certain parameters, which are equal to a boundary of the parameter restrictions. This may imply that the local optimizer used by the benchmark implementation ended up in a different local minimum on the respective trading days, leading to a compensation of the high $\theta$ value by extreme settings for other parameters. As the parameters in the TS model are not completely “independent”, in the sense that different combinations of parameter values may result in the more or less same calibration loss, we achieve much less fluctuating parameters while maintaining a similar calibration result. This can be seen for example in the period around July 2019, where we observe simultaneous peaks respectively lows in $\theta$ and $\gamma$ values, whereas our parameter values are more or less stable trough this period.

A similar issue can be observed for the parameter $\kappa$. In the period from September 2019 to mid January 2020, the estimated parameter of the benchmark implementation starts with values from 0.777 to 2.11 in early September, decrease to 0.56 mid September and then plumbs to 0.04 in mid January 2020 and increases sharply afterwards to 2.5 in spring 2020. In contrast, the CaNN parameter fluctuates from September 2019 with values around 1.5 to end of January 2020 with values of 1.07 with considerably less fluctuations within this period. The same behavior can be observed for $\sigma$ in the aforementioned time period. The evolution of CaNN estimates for different parameters show significantly lower fluctuation and that the parameters are less likely to take extreme values.

Based on these observations, we conclude that the CaNN framework generally provides more stable parameter estimates over time. From our point of view, the stability of parameter estimates over time is a desirable property of a calibration procedure. The estimated model parameters are not only required as inputs for the pricing function, but also to specify stochastic processes in Monte-Carlo simulations for the purpose of calculating P&L components, such as Credit Valuation Adjustments (CVA), and risk measures. Hence, more stable parameters might significantly contribute to a reduction of day-to-day P&L volatility and costs of hedging in the trading business. Furthermore, more stable calibration results will lead to less volatile and more reliable risk measures, which enables managers to take more profound business decisions. This makes the CaNN approach highly relevant for risk managers of financial institutions.

### Discussion and additional results

In summary, the results of our empirical study give rise to the conjecture that an ANN based calibration framework does not only provide competitive results compared to traditional approaches, but also offers further benefits and advantages with respect to the stability and reliability of resulting parameter values. Hence, we conclude that there is indeed a practical applicability for ANN based calibration frameworks. However, we recognize that the practical application of a CaNN framework might involve challenges with respect to the fulfilment of regulatory requirements. Especially, with respect to risk management there are extensive regulatory requirements for the application of internal models (e.g. ECB 2019; OCC et al. 2011). Amongst others, the European Central Bank’s guide on internal models (ECB 2019) introduces regulatory requirements and expectations for the validation of pricing functions and calibration procedures. As an example, ECB (2019) defines a pricing function in the context of an internal Counterparty Credit Risk (CCR) model as the dedicated implementation of a pricing model also taking into account its method for calibration. Furthermore, it requires the inclusion of pricing functions used for calculating or calibrating exposure methods into the model’s framework and governance. Based on this definition, institutions are required to implement a framework that allows for a granular identification of pricing deficiencies (on transaction level). According to ECB (2019) the validation framework needs to include all pricing functions used in the internal model. Hence, we argue that methods and pricing functions used for calibration are subject to the same requirements as pricing functions applied for valuation of derivatives within the exposure simulation.

The proposed calibration framework is a two-step approach, where pricing and calibration are separated. The pricing function is approximated explicitly via an ANN before the actual calibration step. In contrast, a one-step approach calibrates parameters of a dedicated pricing model from market prices directly. Nevertheless, the one-step approach involves an implicit approximation of the model’s pricing function that should be validated according to regulatory requirements. This might be challenging as no explicit pricing function is available in the calibration process and the parameters of the ANN are hard to interpret. In a two-step approach, the validation of the pricing function used within the calibration procedure is straightforward. We are able to identify deviations of ANN prices to the traditional pricing function and market prices on transaction level. Furthermore, the validation of the ANN’s approximation of the pricing function as well as the results of the calibration process can easily be integrated in the validation framework including various materiality thresholds for deviations. Hence, a two-step approach might allow for a straightforward fulfilment of the aforementioned regulatory requirements. In our opinion, the framework proposed in this paper is generally compliant with supervisory expectations as we offer a staggered approach involving additional and separate validation steps 2.2 and 3.2 for the ANN based pricing as well as calibration procedure.

Neural networks are often considered black boxes as it is somewhat difficult to explain and track the mapping function due to the high complexity and high amount of parameters. Hence, regulators may not be fully convinced of a full replacement of traditional calibration frameworks with ANN based calibration procedures. But in contrast to other use cases of machine learning algorithms, such as prediction of future stock returns or risk figures, we know the ground truth of the mapping function we want to approximate, i.e. the TS model. Hence, it is possible to validate our pricing results, in step 2.2, and our calibration results as outlined in step 3.2. These, to some extend unique validation steps of this framework, are strong arguments in the discussion with regulators.

Moreover, we argue that this framework can be utilized to generate initial values for the currently implemented calibration procedures, which should lead to a faster and more robust calibration process. As the initial calibration is performed by calling the ANN, financial institutions are able to reduce dependencies between pricing and calibration procedures in daily production, especially if the solution of the financial model can only be determined by Monte Carlo simulations. Hence, financial institutions could be able to monetize the benefits of ANN based calibration without replacing traditional approaches for now. Based on our results this could increase the stability of results over time and reduce the probability of a local optimizer getting stuck in a local minimum. Additionally, we find that the number of function evaluations required for the local optimizer can be reduced by more than one third using the start values obtained from the CaNN calibration instead of values of the previous day. We are able to provide empirical evidence for the latter aspect in the following case study, where we repeat the calibration process of Sect. 4.3 in two different settings. In the first setting we only use the local Levenberg–Marquardt (LM) optimization algorithm (see Levenberg 1944; Marquardt 1963) to calibrate the parameters. In the second setting, we first use the differential evolution algorithm and *afterwards* pass these values to the LM optimization as initial values. We measure the performance over the out-of-time period based on the function evaluations required by the LM algorithm to arrive at the optimum on each day. Both optimizations are performed in the CaNN framework and on the same hardware to ensure comparability. On average the stand-alone LM algorithm (with previous day start values) requires 253 evaluations per trading day, while the combined optimization only requires 161 evaluations. Hence, we were able to decrease the number of function evaluations by about 36%, while keeping the level of accuracy. This is a considerable reduction leading to a faster calibration process and reduces the computational capacities required and additionally lead to more robust parameter values over time. Furthermore, it is a cheap and efficient way for financial institutions to use a global optimizer, without altering their actual calibration framework. The generation of the daily start values with the DE algorithm does not take longer than 30 s, which probably is considerably less than the potential speed up due to less function evaluations. These results support our conclusion that the implementation of a CaNN framework provides added value, even if traditional calibration procedures are not fully replaced yet.

## Conclusion

This paper provides the first comprehensive proof of concept regarding the practical application of artificial neural networks (ANNs) for the calibration of asset pricing models. We propose additional steps for the CaNN framework based on Liu et al. (2019) to accelerate practical applicability and counteract regulatory concerns for the practical implementation. First, we provide a blended concept for the generation of train and test data. Second, we introduce additional validation procedures based on real-life historic market data to ensure that results of the CaNN are conform with observed pricing and calibration results. Third, we perform a real out-of-time validation to provide evidence that the CaNN framework can cope with unseen data.

Based on a comprehensive time series of historic market data, we are able to show that the calibration framework produces competitive calibration results for a complex IR term structure model compared to a benchmark implementation of a large financial institution. Our empirical analysis covers 1.75 years of swaption data, including the stressed market environment following the break-out of the COVID-19 pandemic. Hence, the calibration approach is suitable for real-life calibration problems and the CaNN framework performs well in different market environments. Given the substantial acceleration of the calibration process by using the CaNN framework, the efficient application of a global optimizer is feasible. As shown in the empirical analysis, the global optimizer is less likely to adopt boundary solutions, leading to more stable parameter results over time compared to the benchmark implementation. At the same time the CaNN framework is able to cope with changing market environments, while maintaining a comparable level of calibration error. The more stable parameter estimates from the CaNN framework might help to reduce the P&L volatility over time, while still ensuring that the model is consistent with the risk-neutral expectations of market participants. Hence, a CaNN framework will provided added value, beyond a potential acceleration of the calibration process. The assessment of the potential benefit with respect to P&L volatility is complex and subject to further analysis.

Further conclusions for the practical implementation of an ANN based calibration framework are as follows. First, the composition and quality of train and test data is a major driver of the CaNN’s performance. Historic swaption data should not be used for training and testing as the data is more valuable for validation. Hence, we propose a blended approach, which produces synthetic data by combining information from historic market data with an algorithm that simulates synthetic datasets. Second, we recommend to set start values for the global optimizer based on the previous day’s results as this significantly accelerates the CaNN calibration process.

We are aware that our empirical analysis is limited to one IR term structure model for a single currency (EUR). The decision to use the Trolle–Schwartz model was based on the aspiration to analyze the performance of the calibration framework for a rather complex, but practically implemented model. Hence, this is the first study to investigate whether ANNs are faster and more robust compared to an implementation of a large financial institution. Furthermore, the TS model can be easily reduced to more simplistic term structure models. However, we believe that the application of this framework to further currencies, models and asset classes will provide further findings regarding the performance of ANN based calibration frameworks. Future work may also focus on obtaining additional insights with respect to the calibration procedure from the CaNN framework, such as information on parameter sensitivity or importance of different inputs. Although we believe that the framework generally adheres to regulatory requirements, its practical application might be viewed critical by supervisory authorities as the training process and resulting ANN pricing function may seen as not fully traceable. To counteract this, we offer a staggered approach involving additional and separate validation steps for the ANN based pricing as well as calibration procedure. However, regulators might still have concerns about the replacement of traditional implementations with the CaNN framework. Nevertheless, the implementation of this framework and the subsequent integration of its results could significantly improve traditional calibration procedures in terms of accuracy, robustness, speed and provide additional insights for validation processes. These aspects give rise to the conjecture that the CaNN framework is of high practical relevance and has the potential to improve model calibration, risk assessment and business decisions.

## Footnotes

1 Ruf and Wang (2020) provide a comprehensive review of literature on the application of neural networks for option pricing and hedging.

2 Horvath et al. (2021) train a neural network on a financial model in a first step and use this for the calibration in an consecutive step. The main difference between Liu et al. (2019) and Horvath et al. (2021) is the type of neural network employed. The latter authors use convolutional neural networks (CNN) as they focus on a 2-dimensional volatility surface, which can be interpreted as a picture. This enables Horvath et al. (2021) to lift all potentials of the CNN proved for pattern recognition and processing of pictures. In our empirical application, we use a financial model, where the volatility surface/prices are represented by 3-dimensions. As the transfer of the output layer of a CNN to higher dimensions is not trivial, we employ a feed-forward neural network similar to Liu et al. (2019). Hence, we follow the calibration framework of Liu et al. (2019) more closely than Horvath et al. (2021).

3 Within this paper we provide an overview of the Trolle–Schwartz model based on Trolle and Schwartz (2009). Hence, we do not provide mathematical derivation, proofs and background of the model. For additional information on the model and its methodological foundations, please refer to Trolle and Schwartz (2009) and Kienitz et al. (2020).

4 For additional details and background on the pricing of swaptions under the TS model, please refer to Trolle and Schwartz (2009) and Kienitz et al. (2020).

5 We are aware that Trolle and Schwartz (2009) propose to use more dimensions. However, our focus is not TS model and its practical implementation. Our paper tries to provide evidence whether an implementation at a financial institution can be substituted or accelerated by an ANN calibration framework. Hence, we follow exactly the setup of the given benchmark to get a reliable and adequate comparison. Therefore, we have to choose $N=1$ dimensions. We thank participants of the 9th International Conference on Futures and Other Derivatives (ICFOD) 2020 and the 33rd Australasian Finance and Banking Conference (AFBC) 2020 for putting emphasis on that point.

6 In the original paper of the CaNN framework by Liu et al. (2019) a penalty term of $10 \cdot 10^{-6}$ is added to the calibration loss to avoid overfitting. They used 35 instruments per calibration task and determined five parameters in the Heston model and eight parameters in the Bates model. Hence, the number of instruments is higher than the parameters to calibrate, but the ratio is lower than in our application. In the empirical section, we calibrate the seven parameters of the TS model on approxmately 800 swaptions per calibration task.

7 In addition, there are discussions to use ANNs trained on market data for pricing and calibration without using a traditional pricing model at all. While this method could theoretically provide a better fit to market data, it imposes several issues with respect to its lack of traceability and the arbitrary choice of the ANN’s properties (i.e., number of parameter, feature selection). Furthermore, evidence for the stability and robustness in practical applications of these approaches still need to be provided.

8 Within this paper we provide a short overview on the mathematical foundations of ANNs only. For a comprehensive summary of the most common mathematical concepts of deep learning, please refer to Kraus et al. (2020).

9 We use 7.68 million swaptions as training data to estimate roughly 2.9 million parameters of the ANN.

10 We also investigated different variants of the loss functions, such as the mean absolute error (MAE), the mean absolute precentage error (MAPE) and an inverse weighting scheme, where we multiply the squared differences by a scaling factor of $\frac{1}{p_j^{(model)}}$ to put more weight on small prices, but find no superior performance in the calibration task.

11 Please note that we use the default values in the implementation of the Python package *SciPy*, except for the population size which we set to 49.

12 We use Intel Core i7-9700 CPU cores with 3.00 GHz.

13 We use the historically observed yield curves for the following currencies: EUR, USD, GBP, JPY, CHF, DKK, NOK, SEK

14 In unreported results, we employed a hyper parameter search only for ATM options, and found the same tendency towards more complex and deeper neural networks. This gives rise to the conjecture that the complexity of the ANN is by a large part determined by the complexity of the IR dynamics, and the number of instruments plays only a minor role. This is plausible, as all instruments share the same yield curve and TS model parameters at a specific trading day, which accounts for a large number of the input parameters.

15 In both calibration frameworks the respective calibrated parameter values of the previous day are used as starting values for the next day. Hence, the more stable results of the CaNN approach may not be attributed to the way the staring values are set.
